# Reduction of Traumatic Brain Damage by Tspo Ligand Etifoxine

**DOI:** 10.3390/ijms20112639

**Published:** 2019-05-29

**Authors:** Mona Shehadeh, Eilam Palzur, Liat Apel, Jean Francois Soustiel

**Affiliations:** 1 Eliachar Research Laboratory, Galilee Medical Center, P.O. Box 21, Nahariya 2210001, Israel; monas@gmc.gov.il (M.S.); eilamp@gmc.gov.il (E.P.); 2 Institute of Pathology, Galilee Medical Center, P.O. Box 21, Nahariya 2210001, Israel; Liata@gmc.gov.il; 3 The Azrieli Faculty of Medicine in the Galilee, Bar Ilan University, Safed 13100, Israel; 4 Department of Neurosurgery, Galilee Medical Center, P.O. Box 21, Nahariya 2210001, Israel

**Keywords:** mitochondria, traumatic brain injury, mitochondrial permeability transition pore, translocator protein, etifoxine

## Abstract

Experimental studies have shown that ligands of the 18 kDa translocator protein can reduce neuronal damage induced by traumatic brain injury by protecting mitochondria and preventing metabolic crisis. Etifoxine, an anxiolytic drug and 18 kDa translocator protein ligand, has shown beneficial effects in the models of peripheral nerve neuropathy. The present study investigates the potential effect of etifoxine as a neuroprotective agent in traumatic brain injury (TBI). For this purpose, the effect of etifoxine on lesion volume and modified neurological severity score at 4 weeks was tested in Sprague–Dawley adult male rats submitted to cortical impact contusion. Effects of etifoxine treatment on neuronal survival and apoptosis were also assessed by immune stains in the perilesional area. Etifoxine induced a significant reduction in the lesion volume compared to nontreated animals in a dose-dependent fashion with a similar effect on neurological outcome at four weeks that correlated with enhanced neuron survival and reduced apoptotic activity. These results are consistent with the neuroprotective effect of etifoxine in TBI that may justify further translational research.

## 1. Introduction

Mitochondrial permeability transition has emerged during the past two decades as a pivotal event in the process of neuronal death in a vast range of neurological pathologies including stroke, neurotrauma, as well as several neurodegenerative conditions including Parkinson’s and Alzheimer’s diseases. During this process, the cyclophilin D combines with the adenine nucleotide translocator (ANT) to form a high conductance channel, the mitochondrial permeability transition pore (mPTP), across the inner mitochondrial membrane (IMM), otherwise impermeable under normal conditions, in contrast to the outer mitochondrial membrane (OMM) [1]. Opening of mPTP, in turn, results in a nonselective traffic of water and small molecules up to 1.5 kDa, leading to the loss of the proton gradient with subsequent membrane depolarization essential for activation of the ATP synthase. As IMM permeabilization develops, mitochondrial matrix swells resulting eventually in a rupture of the OMM with the release of pro-apoptotic proteins into the cytosol such as, cytochrome c and apoptosis inducing factor (AIF). Although the exact structure of the mPTP remains a matter of controversies, several molecules have been advocated as possible regulators of the pore activity. Among these, the 18 kDa translocator protein (TSPO) has gained increasing attention initially, triggered by its co-immunoprecipitation with the ANT and the voltage dependent anion channel (VDAC) and its location at the OMM [2,3]. The hypothesis of a possible regulating role of the TSPO over the process of mitochondrial permeability transition has been since supported by several studies showing an enhanced TSPO expression in several malignant cancer tumors including breast, colon, liver, and brain [4,5,6,7,8,9,10]. Based on these observations, numerous studies have shown that PK11195, a specific TSPO ligand, could enhance apoptosis in various cancer cell lines [11,12,13,14,15]. In most of these studies, PK11195 resulted in enhanced apoptosis [11,12,13,15,16] and even reverse of the antiapoptotic protection mediated by Bcl-2 [14,17]. In contrast, the benzodiazepine Ro5-4864 has been shown to have protective effects in similar conditions [18]. Since PK11195 and Ro5-4864 were initially categorized as antagonist and agonist TSPO ligands [19], it may be hypothesized that Ro5-4864 and additional agonists may be of potential therapeutic interest as neuroprotective agents through inhibition of the mPTP. This hypothesis has been supported by preliminary in vitro studies showing that addition of TSPO ligands to mitochondrial pellets resulted in a protective effect against known mitochondrial noxious agents such as, calcium and Bax, with preserved mitochondrial membrane polarization, reduced release of AIF, and decreased activation of caspase 9 [20,21]. These findings were further supported by in vivo animal studies showing that treatment with Ro5-4864 was associated with enhanced neuronal survival, improved oxidative metabolism expressed by lower lactate/pyruvate ratio, and decreased mitochondrial damage following cortical injury [21,22].

However, the clinical prospective of Ro5-4864 in traumatic brain injury (TBI) remains limited because of established epileptogenic adverse effects [23,24]. In contrast, etifoxine is another TSPO ligand that is currently used in common clinical practice for the management of adjustment disorder with anxiety [25]. Unlike Ro5-4864, etifoxine is not a benzodiazepine but a benzoxazine that nonetheless proved to inhibit the binding of [3H]PK11195 with an IC50 within the micromolar range, resulting in a significant increase in concentrations of pregnenolone, progesterone, 5alpha-dihydroprogesterone, and allopregnanolone in the plasma and brain of treated animals [26], suggestive of a potential neuroprotective effect in neurotrauma [27]. Indeed, preliminary studies have indicated some therapeutic beneficial effects of etifoxine in various neuropathological conditions. These effects have been attributed either to reduced brain edema [28], decreased inflammatory response [29,30], or enhanced tissue repair promotion and regeneration [31,32]. Most of these studies have been focused on peripheral nerve injuries so little is known regarding the potential impact of etifoxine on traumatic brain damage. Accordingly, the purpose of the present study is to investigate the potential effect of etifoxine as a neuroprotective agent in neurotrauma.

## 2. Results

### 2.1. Motor and Behavioral Assessment

**Figure 1:**
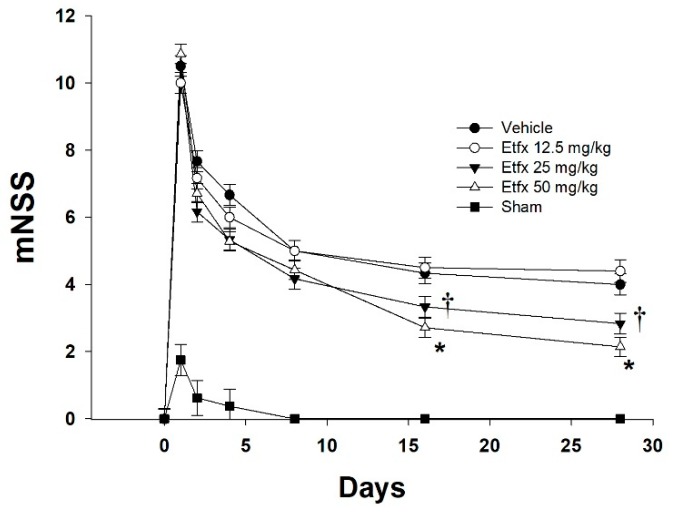
Etifoxine-treated animals had a significantly better functional recovery with higher modified neurological severity score (mNSS) at the end of the follow-up period (4 weeks). This effect of etifoxine (Etfx) on neurological outcome proved to be dose-dependent as only higher doses showed improvement expressed by lower mNSS at day 16 and day 28, whereas a dose of 12.5 mg/kg appeared to be ineffective (repeated measures ANOVA, *p* = 0.0059 effect of group). For each day, mean mNSS values and the standard error of the mean are shown. *: Tukey–Kramer multiple comparison test 50 mg/kg vs. vehicle and 12.5 mg/kg, *p* < 0.05. †: Tukey–Kramer multiple comparison test 25 mg/kg vs. vehicle and 12.5 mg/kg, *p* < 0.05.

Etifoxine-treated animals showed a significant functional improvement expressed by lower mNSS at day 16 and day 28 compared to the vehicle group (Figure 1, repeated measures ANOVA, *p* = 0.0059 main effect of group). This functional positive effect, however, could be observed only in animals treated with higher doses of Etfx (Tukey–Kramer multiple comparison test 25 and 50 mg/kg vs. vehicle, *p* < 0.05), whereas a lower dosage of 12.5 mg/kg proved to be ineffective, with mNSS values similar to that of the vehicle group (Tukey–Kramer multiple comparison test 12.5 mg/kg vs. vehicle, *p* > 0.05). Interestingly, a dosage of 50 mg/kg did not prove to be any more effective than 25 mg/kg for improvement of neurological outcome (Tukey–Kramer multiple comparison test 25 vs. 50 mg/kg, *p* > 0.05).

### 2.2. Lesion Volume

**Figure 2:**
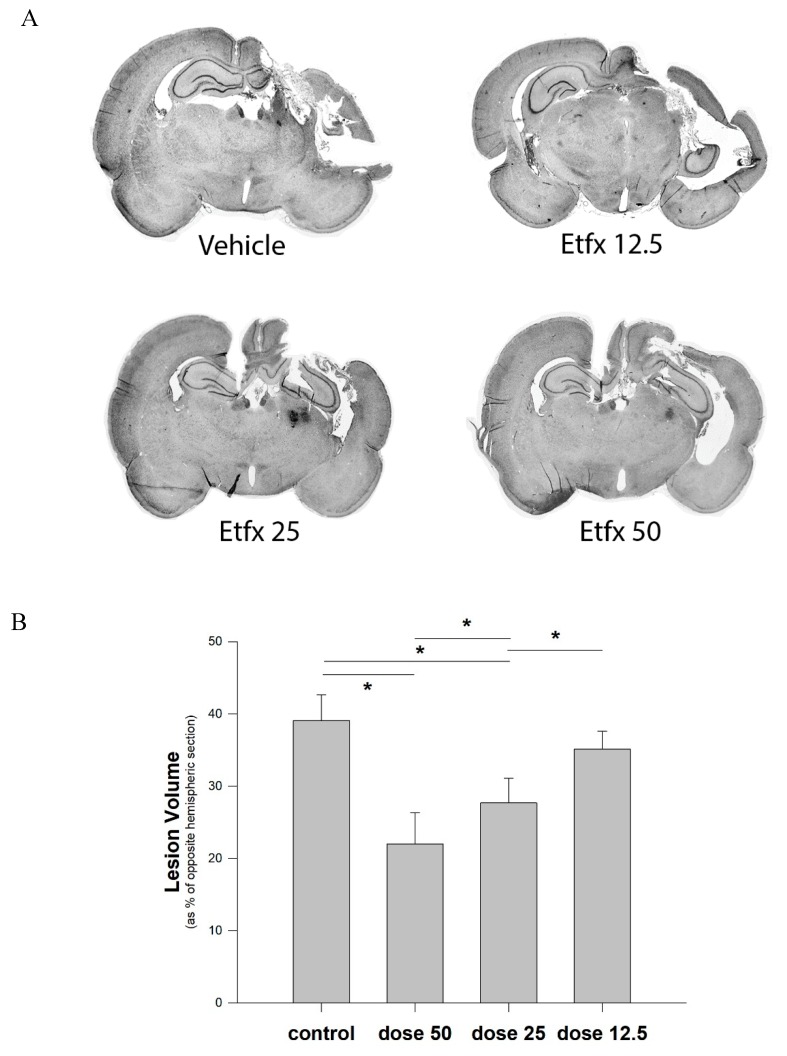
(**A**) The cortical impact injury model created by weight drop magnification micrographs (4×) resulted in an extensive tissue loss in a wide cortical and subcortical area of the injured hemisphere. In contrast, treatment with Etfx significantly reduced the extent of the lesion volume. (**B**) Analysis of the volume of tissue loss (expressed as a percentage of the volume of the brain section on the noninjured side) showed a dose-dependent reduction of the lesion volume at 4 weeks (one-way ANOVA, *p* < 0.001). Bars represent mean lesion volume with the corresponding standard error of the mean. *: Tukey–Kramer multiple comparison *p* < 0.05.

As anticipated, the mixed model used in this study, combining a direct impact to the cortex with the acceleration effect generated by the weight drop on an unfixed head, resulted in a particularly severe injury expressed by a large amount of tissue loss representing close to 40% of the volume of the corresponding brain section in the noninjured hemisphere (Figure 2). 

Analysis of the volume of tissue loss with respect to the opposite uninjured hemisphere showed that high dose Etfx treatment (50 mg/kg) reduced the lesion volume by 44% (39.1 ± 3.6 vs. 22 ± 4.3%, Figure 2, one-way ANOVA, *p* < 0.001—Tukey–Kramer multiple comparison test 50 mg/kg vs. vehicle, *p* < 0.05). Dose reduction was associated with a progressive reduction of the tissue protective effect expressed by significantly higher lesion volumes. As observed with functional outcome, the lowest Etfx dosage (12.5 mg/kg) did not result in any significant reduction of the lesion volume (Tukey–Kramer multiple comparison test 12.5 mg/kg vs. vehicle, *p* > 0.05).

### 2.3. Histological Studies

#### 2.3.1. Neuronal Density

**Figure 3:**
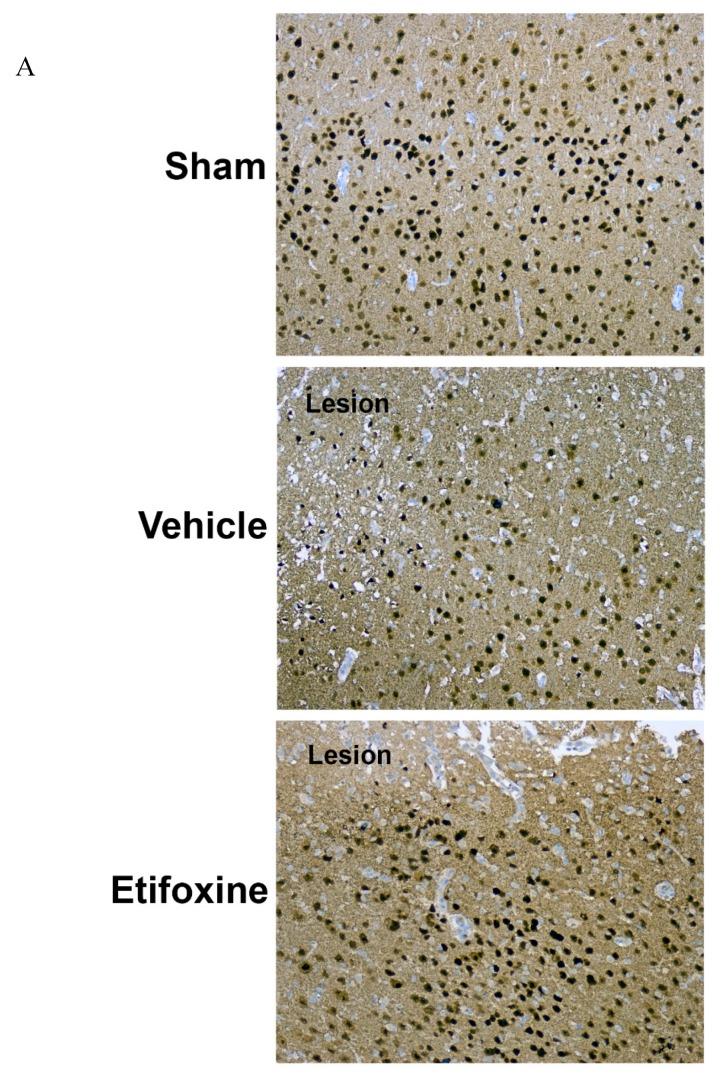

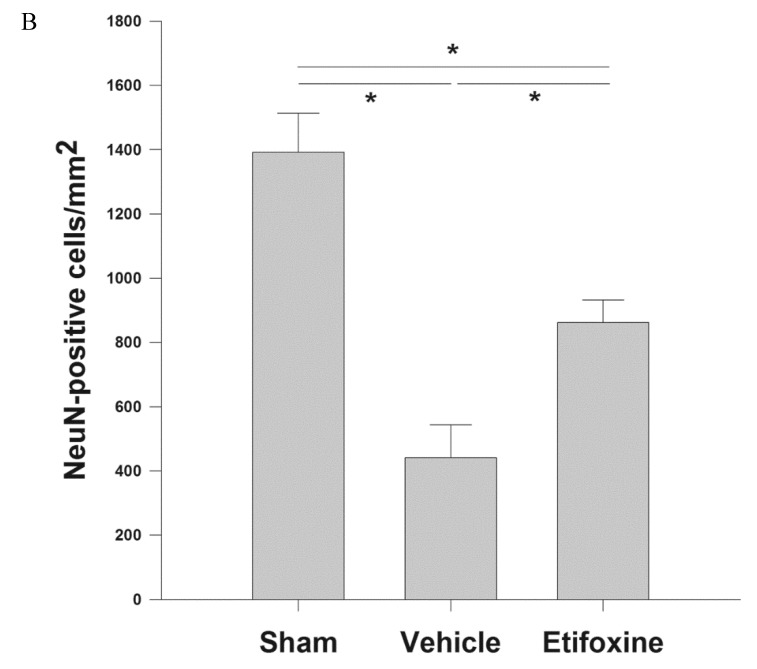
(**A**) Low magnification micrographs (100×) of cortical region stained with anti-NeuN. In comparison with sham animals, cortices of nontreated injured rats were characterized by a marked decreased in neuronal density. Treatment with Etfx was associated with substantially improved neuronal survival. (**B**) Comparison of the neuronal cortical density in the immediate proximity of the lesion core (≤1 mm) in treated and nontreated rats and sham animals. Bars represent mean densities/mm^2^ with the corresponding standard error of the mean. *: one-way ANOVA, *p* < 0.001—Tukey–Kramer multiple comparison test *p* < 0.001.

As a correlate for the large amount of tissue loss, a profound reduction in neuronal density characterized the pericontusional area within the 1 mm perimeter of analysis (Figure 3, one-way ANOVA main effect of group *p* < 0.001, Tukey–Kramer multiple comparison test sham vs. vehicle, *p* < 0.001). This effect on the density of NeuN-positive cells in the perilesional area, however, was markedly and significantly reduced by treatment with Etfx (Tukey–Kramer multiple comparison test Etfx vs. vehicle, *p* < 0.001). 

#### 2.3.2. Caspase-3 Activity

**Figure 4:**
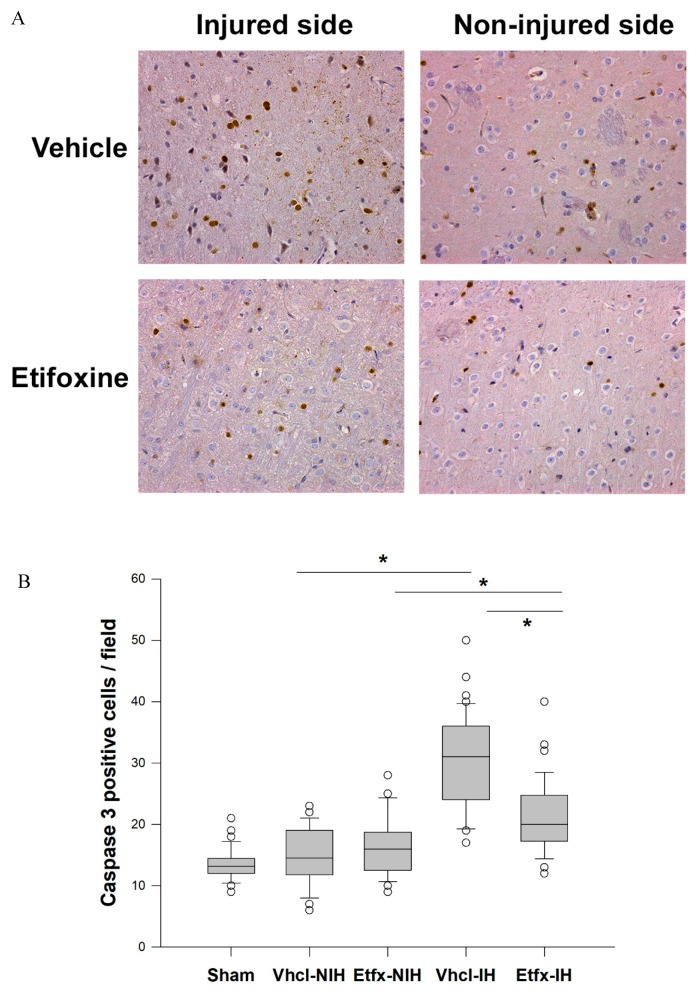
(**A**) In animals of the vehicle group, immunostains for active caspase3 were characterized by a number of caspase-3-positive cells much higher in the perilesional area than in the corresponding cortical region of the opposite noninjured hemisphere. In contrast, the number of caspase-3-positive cells in Etfx-treated animals was profoundly reduced around the injury core and only moderately elevated compared to the noninjured side. Magnification ×200. (**B**) Results of Kruskal–Wallis one-way ANOVA on ranks main effect of group (*p* < 0.001) with Dunn’s multiple comparison test (*: *p* < 0.05).

Expectedly, the number of caspase-3-postive cells was markedly and significantly higher in the perilesional area compared to the opposite noninjured hemisphere in animals of the vehicle group (Figure 4, Kruskal–Wallis one-way ANOVA on ranks main effect of group *p* < 0.001, Dunn’s multiple comparison test vehicle injured vs. noninjured hemisphere, *p* < 0.05). In contrast, treatment with Etfx was associated with a significant reduction of caspase-3 activity around the injury site (Dunn’s multiple comparison test vehicle vs. Etfx on injured sides, *p* < 0.05).

## 3. Discussion

Numerous studies have shown that metabolic crisis and neuronal death in neurotrauma were the consequences of mitochondrial membrane permeabilization through induction of the mPTP. Consequently, mitochondrial damage has emerged as a pivotal event in the neurodegenerative cascade following TBI, being linked to several clinical events including post-traumatic delirium. Interestingly, drugs used in the management of this condition, such as benzodiazepine, have demonstrated remarkable neuroprotective potential toward both excitotoxic and oxidative stress [33]. Direct evidence for involvement of the TSPO in mPTP activity was provided by investigation of the effect of various benzodiazepine ligands on a calcium-dependent and cyclosporine A-sensitive multiple conductance channel [2], previously shown to assume similar properties to that of the mPTP [34]. In this study, mPTP activation was inhibited by TSPO ligands Ro5-4864 and PK11195 but not by the central benzodiazepine ligand clonazepam, which has a known poor affinity for the TSPO [2]. Recently, addition of a specific anti-TSPO antibody in isolated intact rat brain mitochondria resulted in delayed calcium-induced dissipation of mitochondrial transmembrane potential (ΔΨ_m_) and diminished cyclosporine A-sensitive calcium efflux, which are both indicative of mPTP inhibition [20]. The observations made in vitro have been since supported by the findings of animal studies based on the use of TSPO ligands in an experimental TBI model. In this setting, treatment with Ro5-4864 resulted in enhanced neuronal survival, reduced tissue loss, and improved neurological outcome. Importantly, this neuroprotective effect could be attributed to a mitochondrial protective effect expressed by preserved ΔΨ_m_ in treated animals, improved oxidative metabolism and reduced mitochondrial swelling quantitatively evidenced by electron microscopy [21,22]. All together, these findings strongly support the plausible role played by TSPO as a regulator of the mPTP. However, despite the potential therapeutic implications of these studies, no further clinical development has emerged, mostly as the consequence of the known adverse convulsive effects of Ro5-4864 that prevents its implementation in TBI.

Benzodiazepines, however, do not represent the entire spectrum of TSPO ligands, and numerous compounds have been developed including isoquinoline carboxamides [35], benzothiazepines [36], benzoxazines [37], indol acetamides [38], imidazopyridine acetamides [39], phenoxyphenyl acetamides [40], pyrazolo-pyrimidine acetamides [41], and indol-3-ylglyoxylamides [42]. Several of these are currently used for the treatment of anxiety disorders and may be therefore easily implemented within a translational study. Among these, etifoxine is the progenitor of the benzoxazines. It is a non-benzodiazepine anxiolytic drug that proved to inhibit the binding of [3H]PK11195 with an IC50 within the micromolar range and to induce a significant increase in the concentrations of pregnenolone, progesterone, 5alpha-dihydroprogesterone, and allopregnanolone in plasma and brain of treated animals [26]. This effect on neurosteroid synthesis has prompted several experimental studies showing a protective effect in different animal models of neurological disorders including multiple sclerosis [43], but mostly peripheral neuropathy [31,32,44,45,46]. Despite these encouraging results, etifoxine has been scarcely investigated for the treatment of brain injury, proving to be beneficial for the control and relief of brain edema induced by triethyltin in rats and showing improved functional recovery and reduced neuroinflammation in a rat model of TBI [47]. These findings suggest that etifoxine may have a therapeutic potential in the treatment of TBI in a single study [28].

In the present study, etifoxine proved to significantly alleviate both the morphological and the neurological outcomes of cortical impact contusion, with enhanced motor and behavioral functional status, increased neuronal survival, and profoundly reduced tissue loss one month post-injury. Furthermore, these findings were closely correlated with the dose of etifoxine used. Only doses of 25 and 50 mg/kg proved to be beneficial, whereas 12.5 mg/kg failed to produce any significant protective effect. Remarkably, this observation is in accordance with the results reported by Girard et al. in triethyltin-induced brain edema [28]. Furthermore, considering the fact that a direct consequence of mitochondrial membrane permeabilization is represented by the release in the cytosol of cytochrome c with subsequent caspase-3 activation, the observed reduction of caspase-3 activity shown in etifoxine-treated animals further reinforces the hypothesis according to which TSPO-mediated neuroprotection may be achieved by reducing mitochondrial damage as suggested by previous studies [20,21,22]. 

## 4. Material and Methods

### 4.1. Brain Injury Model

All animal procedures were approved by the Bar-Ilan University Animal Care Committee (code #57-12-2014, December 2014) and were carried out in accordance with the National Institutes of Health Guide for the Care and Use of Laboratory Animals as well as ARRIVE (Animal Research: Reporting In Vivo Experiments guidelines). During the study, the animals were housed in groups of 2–3 rats in a sterilized solid bottom cage with contact bedding under controlled temperature and 12:12 h light/dark cycle. Animals were maintained on standard pellet diet and water supplied ad libitum. All efforts were made to maintain animals suffering to minimum and lower the number of animals used as much as possible.

The model of brain injury used was based on the impact acceleration model described by Marmarou et al. [48] Male Sprague–Dawley rats (250–300 g) were anesthetized by intraperitoneal injection of Equithesin (4 mL/kg of body weight). A diamond burr was used in a circular fashion to create a 4 mm diameter hole without injuring the dura. A stainless steel blunt rivet of 1 cm outer diameter and 2 mm depth was then attached to the skull opening while the head was placed on a foam bed with the rivet centered immediately under the lower end of a plexiglass tube. Through the tube, a 900 g weight was dropped from a 60 cm height down to the rivet. The rivet was then removed, and the scalp was then sutured. This modification of the model described by Marmarou et al. was intended to create a mixed TBI lesion associating both diffuse and focal brain injuries. The rats were allowed to recover from anesthesia in an individual cage, under 12 h light/dark cycles with free access to food and water. No acute mortality was related to the model used.

In addition, an additional group of sham-operated animals was added. In this group, animals were anesthetized prior to surgery that consisted in the same burr hole but without cortical injury.

### 4.2. In Vivo Study

#### 4.2.1. Animal Grouping and Treatment

Animals were allocated into 5 groups of eight rats (N = 8) each as follows: Group 1–dimethyl sulfoxide 1% in saline (DMSO-vehicle); group 2–etifoxine in DMSO (Etfx) delivered at an anxiolytic-like dose of 50 mg/kg (Biocodex, Gentilly, France); group 3–Etfx in DMSO 25 mg/kg; group 4–Etfx in DMSO 12.5 mg/kg; group 5–sham-operated animals. For all groups 1 to 4, either DMSO-vehicle or DMSO-Etfx was administered intraperitoneally to the rats at 60 min, 8-, and 24-h post-injury. Timing of drug administration for the first dose was set to the shortest possible time post-injury that could be performed in clinical conditions (prehospital model) and then at 8 and 24 for the second and third dose respectively, in order to increase therapeutic effect. In order to assess the possible variations in outcome measures in response to different dosage regimen for the drug, a dose response study was performed based on the functional and morphological outcomes in response to incremental dosage of 12.5, 25, and 50 mg/kg of etifoxine. The maximal dosage used in this study was based on a pervious study showing a beneficial effect of 50 mg/kg [28]. For each dosage, eight animals (N = 8) were evaluated and their results were compared with that of the vehicle group.

#### 4.2.2. Motor and Behavioral Assessment

Functional performance status was assessed from day 1 to day 28 post-injury, according to the modified neurological severity score (mNSS) by an operator blinded to animal grouping [49]. To rule out a possible behavioral bias in the evaluation, animals were trained on a daily basis for all described tasks prior to surgery and all reached a maximal score.

#### 4.2.3. Lesion Volume

At day 30, the animals were re-anesthetized, and the brains carefully removed, immediately frozen in powdered dry ice and stored at −20 °C. This delayed timing was chosen as both the necrotic area and surrounding edema have cleared, leaving a sharply delineated cavity convenient for unbiased evaluation of the lesion size [22]. Brains were then cut in coronal plane with a cryostat. Slices of 10 µm thick sections were collected every 500 µm from immediately anterior to the lesions to immediately posterior to the lesion and stained with cresyl violet. From the anterior to the posterior limits of the lesion, an average of 9 slices were collected and analyzed. The area of both hemispheres were measured using a computerized image system (Image J software, NIH Image J, National Institutes of Health, Bethesda, MA, USA) by two separate investigators blinded to experimental groups as described previously and the results averaged [50]. The total lesion volume was determined by integrating the area from each section with the distance between slices and expressed as a percentage of the measured volume of the corresponding section of the opposite hemisphere using the following equation: (noninjured section volume − injured section volume)/noninjured section volume.

### 4.3. Immunohistochemistry

#### 4.3.1. Brain Removal and Fixation 

At day 4, rats were deeply anesthetized and transcardially perfused with heparinized saline, 10% sucrose in a buffered saline, and 4% buffered formaldehyde. Brains were then removed from the skull and post-fixed by immersion into 4% buffered formaldehyde for 48 h. Fixed specimens were sectioned through the produced lesion area and embedded in paraffin. Coronal sections of 5 μm thickness were cut through the lesion with a rotary microtome and stained for immunohistochemistry. It should be mentioned that for these histological studies, only a 50 mg/kg dosage of Etfx was used.

#### 4.3.2. NeuN Staining

The monoclonal antibody (mAb) A60 generated against brain cell nuclei recognizes NeuN in most neuronal cell types throughout the nervous system. For NeuN staining, we used anti-NeuN clone A60 monoclonal antibody (Millipore, Temecula, CA, USA). Quantitative assessment was performed in the perilesional area in two different sections randomly cut through the lesion. Five randomly chosen digital microphotographs were obtained from the perilesional area under ×100 magnification and from the corresponding area in the noninjured hemisphere, according to a previously described protocol [21]. Analysis was performed by two independent observers blinded to animal grouping.

#### 4.3.3. Caspase-3

During apoptosis, the caspases, a family of cysteine proteases, play an essential role in the initiation, regulation, and execution of the downstream proteolytic events. One of the primary executioners of apoptosis is caspase-3, which is required for the cleavage of a large number of proteins and for apoptosis-associated chromatin margination, DNA fragmentation, and nuclear collapse. Thus, the detection of activated caspase-3 represents a valuable and specific tool for identifying apoptotic cells in tissue sections, even before all the morphological features of apoptosis take place [51]. For this purpose, we used rabbit anti-active caspase-3 (Zytomed systems, Berlin, Germany). Quantitative assessment was performed in two different noncontiguous sections randomly cut through the lesion, both in the perilesional area and in the corresponding area in the opposite hemisphere. Randomly chosen digital microphotographs of both slices were obtained 200× magnification. At least 10 visual fields in each slice were acquired for averaging the number of caspase-3-positive cells for each hemisphere. Analysis was performed by two independent observers blinded to animal grouping.

### 4.4. Statistical Analysis

Variations in the indices of the different parameters in the different groups were explored by different models of ANOVA (one-way and repeated-measures) or Kruskal–Wallis one-way ANOVA on ranks according to data requirement. Whenever appropriate, post hoc analysis of differences noted between groups were tested using the Tukey–Kramer or Dunn’s multiple comparison procedure. A *p*-value of less than 0.05 was considered significant.

## 5. Conclusions

In conclusion, the results of the present study are consistent with the neuroprotective effect of etifoxine in TBI. Noticeably, our findings are in accordance with that of Simon–O’Brien et al. [47] following experimental TBI. However, the translation of these findings into more advanced stages of clinical research is challenging due to several reasons represented by the complexity of diagnosis, prognosis, and treatment of neurotrauma. First, the spectrum of pathological conditions is broad and state-of-the-art clinical/radiological screening might result in false negatives. Second, due to the involvement of many neural networks, it is difficult to expect a major breakthrough from a pharmacological strategy based on a single agent. Third, the unpredictable impact on specific cognitive functions makes it difficult to forecast long-term functional outcome [52]. Therefore, the need for ongoing laboratory studies, expanding the spectrum of TSPO synthetic ligands seems warranted in order to pave the way for the development of novel neuroprotective strategies.
